# Maspin expression is frequent and correlates with basal markers in triple-negative breast cancer

**DOI:** 10.1186/1746-1596-6-36

**Published:** 2011-04-16

**Authors:** Yoshihisa Umekita, Yasuyo Ohi, Masakazu Souda, Yoshiaki Rai, Yoshiaki Sagara, Yasuaki Sagara, Shugo Tamada, Akihide Tanimoto

**Affiliations:** 1 Department of Molecular and Cellular Pathology, Field of Oncology, Kagoshima University Graduate School of Medical and Dental Sciences 8-35-1, Sakuragaoka, Kagoshima 890-8544, Japan; 2 Hakuaikai Sagara Hospital, Kagoshima, 3-31, Matsubara, Kagoshima 892-0833, Japan

## Abstract

**Background:**

Maspin is a unique member of the serine protease inhibitor superfamily and its expression is found in myoepithelial cells of normal mammary glands; therefore, it has been considered to be a myoepithelial marker. We previously reported that maspin was frequently expressed in biologically aggressive breast cancers. In turn, triple-negative (TN) breast cancer is a subtype of tumor with aggressive clinical behavior and shows frequent expression of basal markers. We hypothesized that maspin expression may be frequent and correlate with basal rather than myoepithelial markers in TN breast cancer.

**Methods:**

Paraffin-embedded 135 TN invasive ductal carcinoma tissue samples were immunohistochemically investigated using the Dako Envision+ kit and primary antibodies for maspin, basal (CK5/6, EGFR, CK14) and myoepithelial markers (p63, CD10). The correlation between maspin expression and relapse-free survival (RFS) was investigated by the log-rank test.

**Results:**

The positive rate for maspin was 85.9% and significantly correlated with younger age (*P *= 0.0015), higher histological grade (*P *= 0.0013), CK5/6 positivity (*P *< 0.0001), CK14 positivity (*P *= 0.0034) and the basal-like subtype defined by CK5/6, EGFR and CK14 positivity (*P *= 0.013). The positive rates for CK5/6, EGFR, CK14, CD10 and p63 were 59.2%, 48.9%, 34.1%, 17.8% and 12.6%, respectively. There was no significant correlation between maspin expression and RFS.

**Conclusions:**

The positive rate for maspin is the highest among known basal and myoepithelial markers, and strongly correlates with basal markers in TN breast cancer. These results suggested that maspin could be a candidate for a therapeutic target for TN breast cancer.

## Introduction

Maspin is a unique member of the serine protease inhibitor superfamily and it has been shown to have tumor suppressive activity attributable to the inhibition of breast cancer cell motility, invasion and metastasis [1]. Its expression is found in myoepithelial cells of normal mammary glands; therefore, it has been considered to be a myoepithelial marker, but its correlation with basal markers, such as CK5/6, CK14 and epidermal growth factor receptor (EGFR), in breast cancers remains to be solved. On the other hand, triple-negative (TN) breast cancer is a subtype of tumor with aggressive clinical behavior which currently lacks effective targeted therapies [2]; however, TN breast cancer encompasses a remarkably heterogeneous group of tumors, and the expression of basal markers identifies biologically and clinically distinctive subgroups of TN tumors [2]. We previously reported that maspin expression was an independent poor prognostic indicator in invasive ductal carcinoma (IDC) [3], and that its expression was up-regulated during the progression of mammary ductal carcinoma [4]. Additionally, Rakha et al. reported that basal, not myoepithelial, phenotypes defined by CK5/6 and/or CK14 positivity had an independent value in predicting a poor clinical outcome in a large number of invasive breast carcinomas [5]. Taken together, we hypothesized that maspin expression could be frequent and correlated with basal rather than myoepithelial markers in TN breast cancer. To explore this hypothesis, we investigated the frequency of maspin expression and its correlation with established basal (CK5/6, EGFR, CK14) and myoepithelial (p63, CD10) markers in TN breast cancer. In addition, we investigated the relationship between maspin expression and relapse-free survival (RFS) in TN breast cancer.

## Materials and methods

Paraffin-embedded tissue samples obtained from 135 TN breast cancer patients between Descember 2001 and March 2006 were collected from Hakuaikai Sagara Hospital (Kagoshima, Japan). All breast cancers were histologically classified as IDC. The median age was 56.6 years (range: 27-91 years). Of 135 patients, follow-up data were obtained from 126 patients. The median follow-up time was 64.2 months (range: 3-136 months). Breast cancer recurred in 27 patients (21.4%) during the follow-up period. All patients, except for one, were histologically examined for axillary lymph node involvement, and 52 patients were histologically diagnosed as node-positive. TN was defined as negative for ER and PgR (cutoff 10%), as well as HER2 negative (Hercep test: score 0, 1+, 2+). In cases that scored 2+, the absence of HER2 gene amplification was confirmed by fluorescent in situ hybridization analysis using the PathVysion kit (Abbott-Vysis, Inc., Downers Grove, IL). Immunohistochemistry was performed using the Dako Envision+ kit in conjunction with the DAKO Autostainer according to the instructions supplied by the manufacturer, as described previously [6]. The primary antibodies used and their cutoff values are shown in Table 1. The basal-like subtype was defined by positive for CK5/6 and/or EGFR [7]. Each staining result was assessed independently by two pathologists (YO and YU). When the evaluations differed, final agreement was reached by consensus. The patients and their tumor characteristics were analyzed using the chi-square test. Actuarial curves for RFS were calculated by the Kaplan-Meier technique. RFS were calculated from the date of first surgery to the date of clinical or pathological relapse. Differences in RFS were tested with the log-rank test. All statistical analyses were performed with a statistical software package (Dr SPSS version 11.0.1J; SPSS Japan Inc., Tokyo, Japan). The cutoff for significance was taken as *P *= 0.05.

**Table 1:** Souce, dilution, pretreatment and cutoff values of primary antibodies used

Antibody (clone)	Manufacturer	Dilution	Pretreatment	Cutoff values
Maspin (EAW24)	Novocastra	1:200	Microwave	≧10% (positive)
CK5/6 (D5/16B4)	Dako	1:50	Water bath	≧10% (positive)
CK14 (LL002)	Novocastra	1:100	Water bath	≧10% (positive)
EGFR (EGFR.113)	Novocastra	1:25	Microwave	≧10% (positive)
CD10 (56C6)	Novocastra	1:50	Water bath	≧10% (positive)
p63 (4A4+Y4A3)	LabVision	1:200	None	≧10% (positive)
ER (1D5)	Dako	1:50	Water bath	≧10% (positive)
PgR (PgR636)	Dako	1:800	Water bath	≧10% (positive)
HER2	Dako	Prediluted (Hercep test)	Water bath	Score 3+ (positive)

## Results

**Figure 1:**
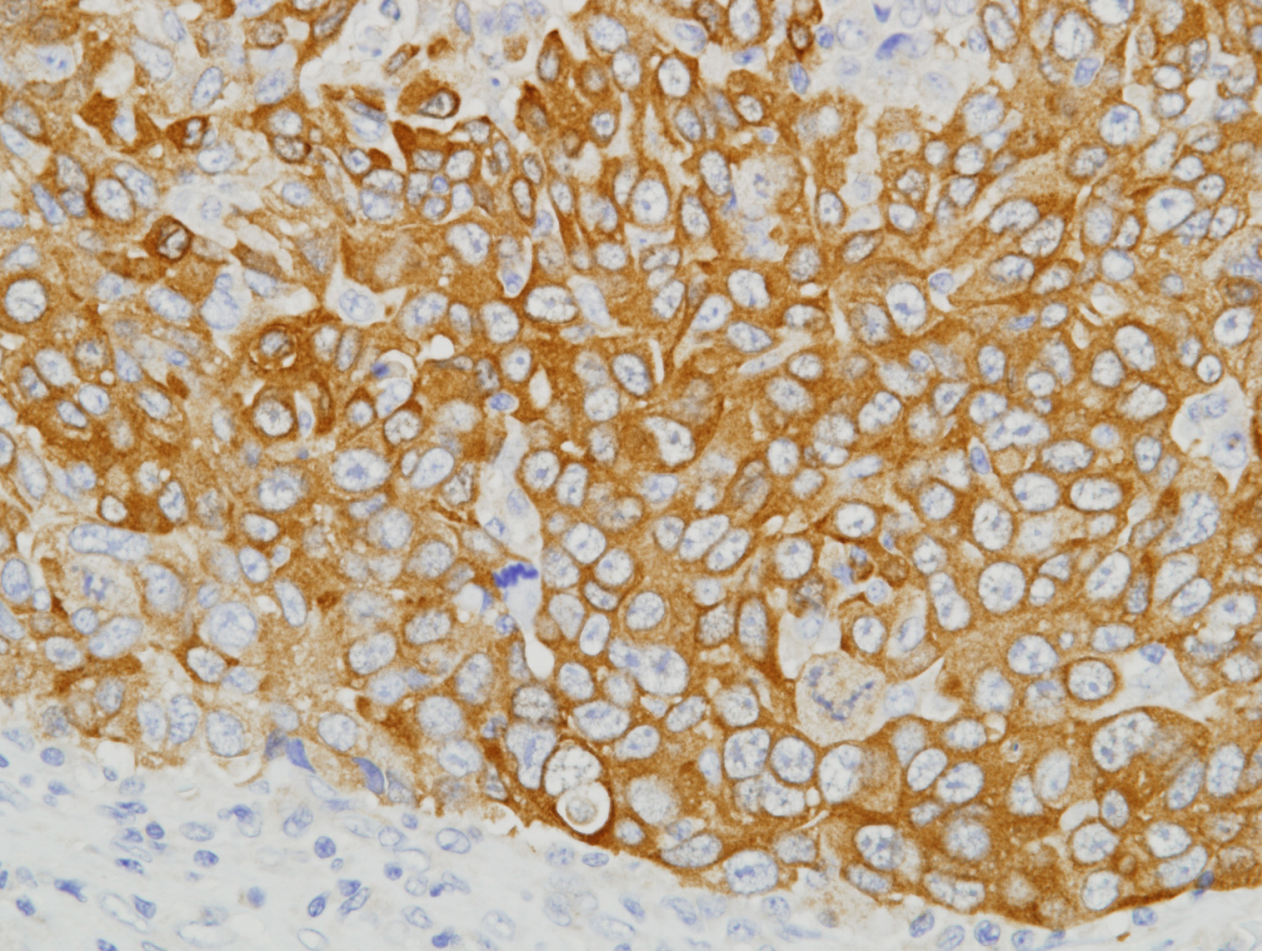
**Immunohistochemical detection of maspin in triple-negative invasive ductal carcinoma**. Cytoplasmic staining was noted in carcinoma cells, and stromal cells were negatively stained.

Patient and tumor characteristics are summarized in Table 2. The positive rates for maspin, EGFR, CK5/6, CK14, CD10 and p63 were 85.9% (116 cases), 48.9% (66 cases), 59.3% (80 cases), 34.1% (46 cases), 17.8% (24 cases) and 12.6% (17 cases), respectively (Table 2). Expression of maspin significantly correlated with younger age (*P *= 0.0015), higher histological grade (*P *= 0.0013), CK5/6 positivity (*P *< 0.0001) and CK14 positivity (*P *= 0.0034) (Table 3). The representative positive staining pattern of maspin is shown in Figure 1. The myoepithelial cells and stromal cells in mammary glands served as internal positive and negative controls, respectively. The basal-like subtype defined by CK5/6 and/or EGFR expression was observed in 76.3% (103 of 135 cases). The positivity for maspin showed a slightly significant correlation with the basal-like subtype (*P *= 0.041) (Table 4). When defined by CK5/6 and/or EGFR and/or CK14 positivity, the basal-like subtype was found in 79.3% (107 of 135 cases) and the positivity for maspin more significantly correlated with the basal-like subtype (*P *= 0.013) (Table 4). The log-rank test showed that only node metastases significantly correlated with RFS (*P *< 0.0001). There was no significant correlation between maspin expression and RFS (*P *= 0.204).

**Table 2:** Patients and tumor characteristics in triple-negative breast cancer cohort

Parameters	Number	Percentage
Age (years)		
≤50	51	37.8
>50	84	62.2
Tumor size (mm)		
≤20	44	32.6
>20	91	67.4
Lymph node metastases		
0	82	60.7
1-3	28	20.7
3<	24	17.8
Histological grade		
I	5	3.7
II	29	21.5
III	101	74.8
Maspin		
Positive	116	85.9
Negative	19	14.1
EGFR		
Positive	66	48.9
Negative	69	51.1
CK5/6		
Positive	80	59.3
Negative	55	40.7
CK14		
Positive	46	34.1
Negative	89	65.9
CD10		
Positive	24	17.8
Negative	111	82.2
p63		
Positive	17	12.6
Negative	118	87.4

**Table 3:** Association between maspin expression and clinicopathological factors in triple-negative breast cancer

	Maspin expression		
		
	Positive (85.9%) 116 cases	Negative (14.1%) 19 cases	*P*-value
Age (years)			
≤50	50	1	0.0015
>50	66	18	
Tumor size (mm)			
≤20	41	3	0.091
>20	75	16	
Lymph node metastases			
0	69	13	0.226
1-3	27	1	
3<	20	4	
Histological grade			
I	4	1	0.0013
II	19	10	
III	93	8	
EGFR			
Positive	57	9	0.886
Negative	59	10	
CK5/6			
Positive	77	3	<0.0001
Negative	39	16	
CK14			
Positive	45	1	0.0034
Negative	71	18	
CD10			
Positive	19	5	0.293
Negative	97	14	
p63			
Positive	15	2	0.769
Negative	101	17	

**Table 4:** Association between maspin expression and basal-like subtype in triple-negative breast cancer

	Maspin expression		
		
	Positive (85.9%) 116 cases	Negative (14.1%) 19 cases	*P*-value
CK5/6 and/or EGFR			
Positive	92	11	0.041
Negative	24	8	
CK5/6 and/or EGFR and/or CK14			
Positive	96	11	0.013
Negative	20	8	

## Discussion

In the current study, we restricted our analysis to IDC of no special type to avoid any confounding effect of special types of invasive breast cancer, such as lobular, medullary and metaplastic carcinomas. Previous DNA microarray and immunohistochemical analyses showed that 80% to 90% of TN breast cancer were basal-like subtypes and had clinical behavior similar to basal-like behavior [2]. The positive rate for maspin in our study was 85.9% of TN breast cancer, and the positive rate was similar to that of basal-like subtypes in TN breast cancer. Although there is no consensus about the definition and method of identification of a basal-like subtype in routine practice, tumors with a basal-like phenotype were strongly associated with a high histological grade in all classifications [2], which is similar to our previous and present findings that maspin positivity strongly correlated with a high histological grade in IDC [3] and TN breast cancer. There is no international consensus on the precise complement of markers that defines a basal-like subtype [2], but Nielsen's definition, used in our study, is currently considered one of the most pragmatic and widely accepted definitions of basal-like breast cancer [7]. The single use of maspin positivity was able to detect 89.3% (92 of 103) of basal-like subtypes defined by Nielsen's definition (CK5/6 and/or EGFR positivity). Including CK14 in Nielsen's definition, 89.7% (96 of 107) of the basal-like subtypes was detected by the single use of maspin. Additionally, all 16 patients that were CK5/6-positive and EGFR-positive, with low histological differentiation and younger age were maspin-positive. Although the analyses of gene expression arrays is required to conclude that maspin is a marker of the basal-like subtype, our results suggested that maspin could be a candidate for a basal marker in TN breast cancer.

It has been hypothesized that maspin may regulate gene transcription in response to cellular stress induced by inflammation, tissue injury and remodeling [8], but its function attributable to an aggressive phenotype in some breast cancers remains to be resolved. We can consider three possible explanations. One is that basal differentiation could contribute to a more aggressive phenotype [5,9]. The second is a high intracellular concentration of maspin resulting in auto-inhibition of its activity by non-covalent polymerization [10]. The third is mutation of the maspin gene causing loss of normal function of the maspin protein. Additionally, it has been reported that two intriguing genes upregulated by maspin re-expression were the E2F transcription factor 1 (E2F1) and the naturally occurring BRCA1 splice variant BRCA1b, and that maspin may play an important role in response to DNA damage at the level of cell-cycle regulation and cellular proliferation [8]. On the other hands, the majority of BRCA1-associated breast cancers is TN and expresses basal cytokeratins [11]. Therefore the elucidation of more precise molecular mechanisms between maspin and BRCA1 may be one of the important targets of future research. It could be argued that instead of identifying descriptive and prognostic molecular subgroups, such as basal-like within TN breast cancer, it would be more clinically relevant to identify patients whose TN tumors are sensitive to specific chemotherapy agents and targeted therapies [12]. It is important for the development of targeted therapies to elucidate the role and function of maspin in TN breast cancer.

## Competing interests

The authors declare that they have no competing interests.

## Authors' contributions

YU designed the study, evaluated immunohistochemistry, performed statistical analysis and wrote the manuscript. YO interpreted the data and evaluated immunohistochemistry. MS performed immunohistochemical experiments. YR, YS, YS and ST participated in the sampling of clinical data. AT performed critical reading of manuscript and supervision. All authors have read and approved the final manuscript.
